# Porous POSS-PANI nanofibre from interfacial polymerization and hydrothermal approach

**DOI:** 10.1186/s40064-015-1524-3

**Published:** 2015-11-25

**Authors:** Lei Liu, Xiaoxuan Xu

**Affiliations:** School of Mechanical Engineering and Suzhou Research Institute, Southeast University, 210096 Nanjing, People’s Republic of China; Zhengde Polytechnic College, 211106 Nanjing, People’s Republic of China

**Keywords:** Polymeric composites, Microstructure, Thermal properties, Hydrothermal

## Abstract

Nowadays, novel applications for polyaniline (PANI) make new demands on its morphology controlling, and designing novel PANI or PANI composite polymeric materials has been more and more attractive. In this work, octaaminophenyl polyhedral oligomeric silsesquioxane (POSS) was employed to prepare nanostructured PANI composites via controlled fabrication. By interfacial copolymerization, fibrous nanostructure was obtained. The size and morphology of this structure was adjusted by changing POSS to OAPS ratio: the size increased from about 20 to 200 nm when the molar ratio of POSS in the composites increased from 0.5 to 2.0 mol %. More importantly, further hydrothermal treatment for the samples with higher POSS concentration resulted in mesoporous structure on a more microscopic scale, which helps to improve the thermal stability. In the total synthesis, POSS played an important role in the morphology controlling of the composites.

## Background

As one kind of conjugated polymers, PANI can be easily obtained via oxidative polymerization in various organic solvents or aqueous system. Because of its good environmental stability under normal processing conditions, excellent physical and chemical properties, adequate level of electrical conductivity and unique doping mechanism, it has been incorporated more and more widely in many applications (Dhand et al. 2010), such as batteries (Li et al. 2014), electro-magnetic interference (EMI) shielding (Teotia et al. 2014) and solar cells (Han et al. 2014). Recent research work has shown that nanostructured PANI can play an important role in sensor applications for its greater sensitivity and faster response time due to its higher effective surface area and shorter penetration depth for target molecules. The developments of PANI in molecular sensors have made new demands on its morphology controlling. Existed work shows that PANI based micro/nanostructures with different dimensions and morphologies can be obtained by template methods (Vijayakumar et al. 2013; Hu et al. 2012), template free methods (Du et al. 2008) and electro-spinning technology (Wanna et al. 2006). For example, zero-dimensional and one-dimensional nanostructures of polyaniline (PANI) can be achieved by using swollen liquid crystals as “soft” templates (Dutt and Siril 2014). PANI micro/nanostructures featuring square nanosheets, microspheres and microdisks are successfully synthesized via hydrothermal method (Zhu et al. 2015). By taking advantage of a microfluidic technology and employing organic soluble acid labile t-Boc-protected PANI as precursor, fabrication of PANI microfibres in a size-controlled manner is possible (Yoo et al. 2015).

On the other hand, the synthesis, characterization and application nanoporous or mesoporous materials have been more and more important in material research field due to their attractive features such as high pore volume, large surface area. Among lots of members in mesoporous family, silica based nanoporous or mesoporous materials have been widely studied for their pore size distribution, regular pore structure and other attractive properties. The can be used in catalyst, separation and purification, drug delivery, gas sensing and other fields. Generally speaking, typical silica based mesoporous materials, such as hexagonal MCM-41, cubic MCM-48, lamellar M41S, and cubic octamer [(CTAB)SiO_2.5_]_8_, can be prepared by silica and surfactant via template-directed synthesis following co-operative assembly pathways. In these preparations, surfactant works as soft template to induce the self-assembly, while silica works as framework in building mesoporous structure. The recent development of silicon chemistry provides us novel choice for selecting silica based framework, such as POSS. POSS are one type of hybrid materials with the general formula (RSiO_3/2_)_n_, whose organic substituents are connected with a silicon-oxygen core. The sizes POSS cages range from 1 to 3 nm, which can be regarded as the smallest possible units of silica (Agaskar and Klemperer 1995; Anderson et al. 2006; Li et al. 2001). Obviously, certain POSS cages with special substituted groups can be used to design novel materials with defined mesoporous structure. In this work, we introduce octaaminophenyl POSS into PANI chains to get nanostructured PANI composites with improved properties by controlled fabrication.

## Results and discussion

### Synthesis of OPS and OAPS

FTIR, NMR and MALDI-TOF characterizations and for OPS are listed as followings: FTIR (cm^−1^) with KBr powder: 3072 (H-Ar), 1594 (C–C), 1112 (Si–O–Si); ^29^Si NMR (ppm): −76.5, MALDI-TOF (m/z): 77, 399, 400, 401, 876, 877, 878, 954, 955, 956, 1031, 1032, 1033 [C_48_H_40_Si_8_O_12_ = 1032 amu].

FTIR and NMR characterizations for OAPS are listed as followings: FTIR (cm^−1^) with KBr powder: 1134 (Si–O–Si), 3383 (N–H), 1488 (C–N), ^29^Si NMR (ppm): −73.3, −77.4; ^1^H NMR (ppm): 7.8–6.2 (4.0H), 5.2–3.7 (2.0H).

**Fig. 1:**
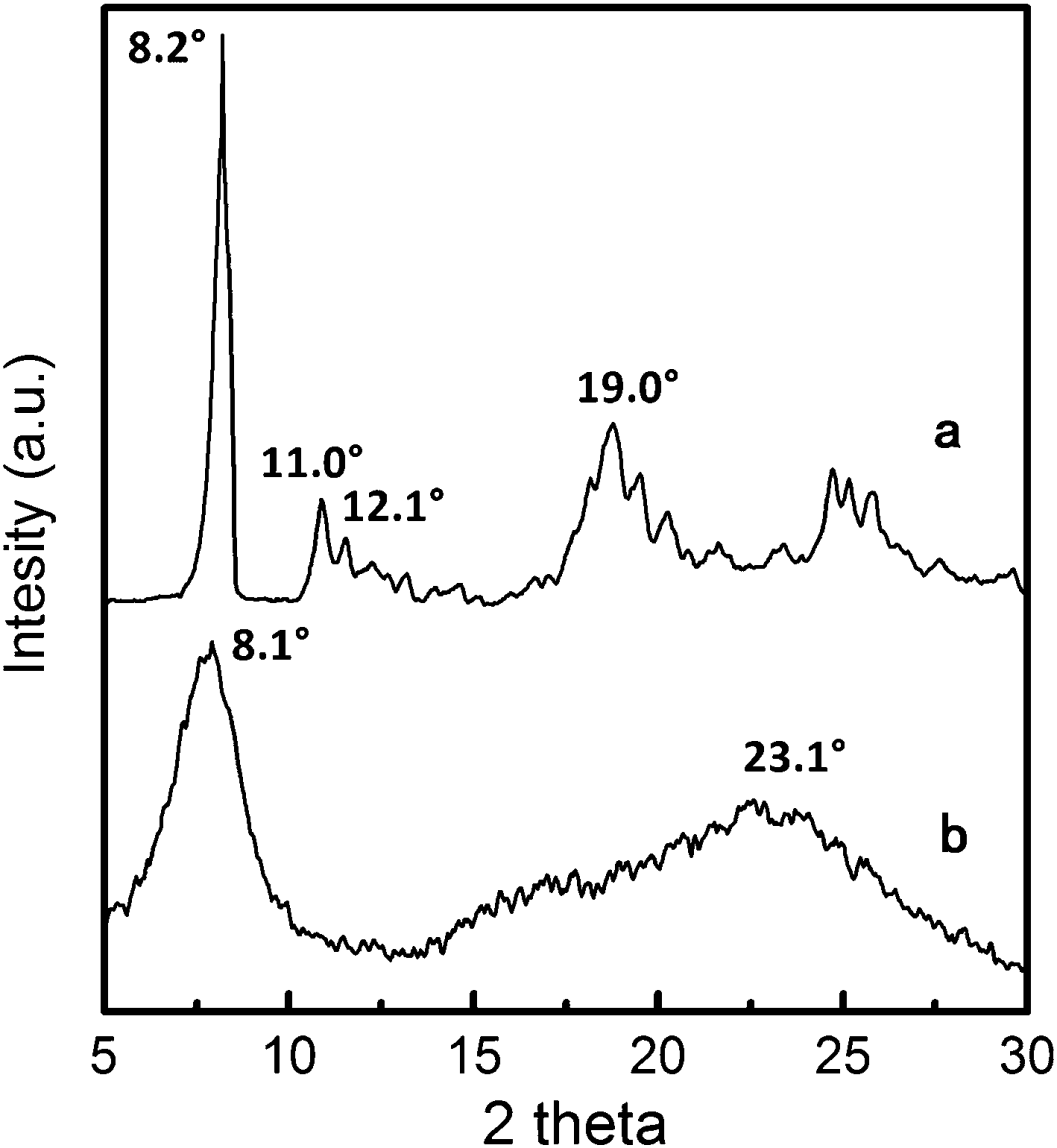
XRD patterns for OPS (*a*) and OAPS (*b*)

XRD pattern for **OPS** (Fig. 1a) shows four strong reflections at 8.2°, 11.0°, 12.1°, and 19.0° correspond to lattice spacings of 10.8, 8.08, 7.35, and 4.73 Å, respectively, while the pattern for OAPS (Fig. 1b) only gives two wide peaks at about 8.1° and 23.1°. The diffraction peak at 8.1° can be explained by some long-range order in solid OAPS, and the broad peak at 23.1° may be associated with Si–O–Si linkages. This indicates the crystal structure of OPS has been destroyed after nitrification and amination. According to the previous research, POSS nanoparticles can form three-dimensional crystal structure by arranging each POSS in a plane on a hexagonal array. During the nitrification and amination of OPS, exotic atoms or substituents are introduced into the phenyls of OPS, but the exact location and number of atom or substituent in each phenyl are not quite sure. Since the different degree of nitrification and amination, there will be differences in the substituents of OAPS, which will change the original stacking sequence and destroy the lattice structure.

### Fibrous POSS-PANI composites

**Fig. 2:**
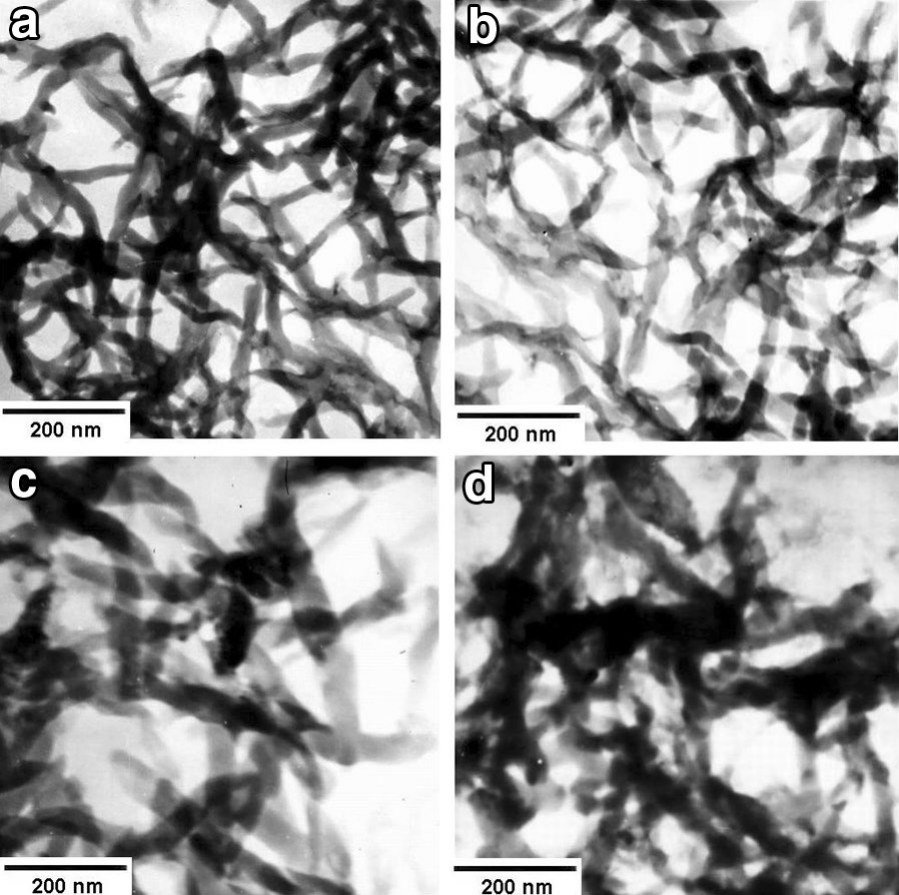
TEM images of POPA-1 (**a**), POPA-2 (**b**), POPA-3 (**c**), and POPA-4 (**d**)

Fibrous nanostructures of POPA-1, POPA-2, POPA-3 and POPA-4 (their detailed composition can be found in Table 1) are showed in Fig. 2a, b, c and d respectively. When POSS concentrations are relative lower (POPA-1 and POPA-2), nanofibre structure with the diameter of 20 to 30 nm can be obtained (Fig. 2a, b). With POSS concentration increasing to a certain mounts (POPA-3), the diameter of nanofibre is increased to 50 nm or so (Fig. 2c). If POSS concentration continues to increase, the diameter of nanofibre goes on to increase, accompanied with partial aggregations, as showed in Fig. 2d. These results indicate that the uniform of the fibrous nanostructure will be decreased and the diameter will be increased with POSS concentration rising. These changes in morphology of composites attribute to their special formation process: at the very beginning of copolymerization, there is few heteronuclei available in the solution, and homogeneous nucleation predominates at early stage, leading to the formation of fibrous nanostructures. The polymerization of aniline is always accompanied by precipitation, and small molecular aggregations are formed when their concentration exceeds a specific level. On the other hand, the existence of POSS will change the pathway of polymerization. OAPS possesses eight aniline groups at each corner of the cubic cage. It can be linked by aniline to form PANI-tethered POSS and thus forms POSS-PANI composites. Multifunctional POSS cages lead the polymerization reaction towards branching in eight directions, which is not advantageous to the linear growth of PANI chains. Comparing Fig. 2c, d to a, b, it can be found that the linear parts of nanofibre are shortened, and the diameters of nanofibre increase with POSS concentration increasing.

**Table 1:** Detailed POSS-PANI composite samples

Sample ID	OAPS concentration (mol %)	Details
POPA-1	0.5	Interfacial polymerization, 25 °C, pH = 3.0
POPA-2	1.0	
POPA-3	1.5	
POPA-4	2.0	
POPA-5	2.0	24-h hydrothermal treatment
POPA-6	2.0	48-h hydrothermal treatment
POPA-7	2.0	96-h hydrothermal treatment

### Porous POSS-PANI composite fibres

**Fig. 3:**
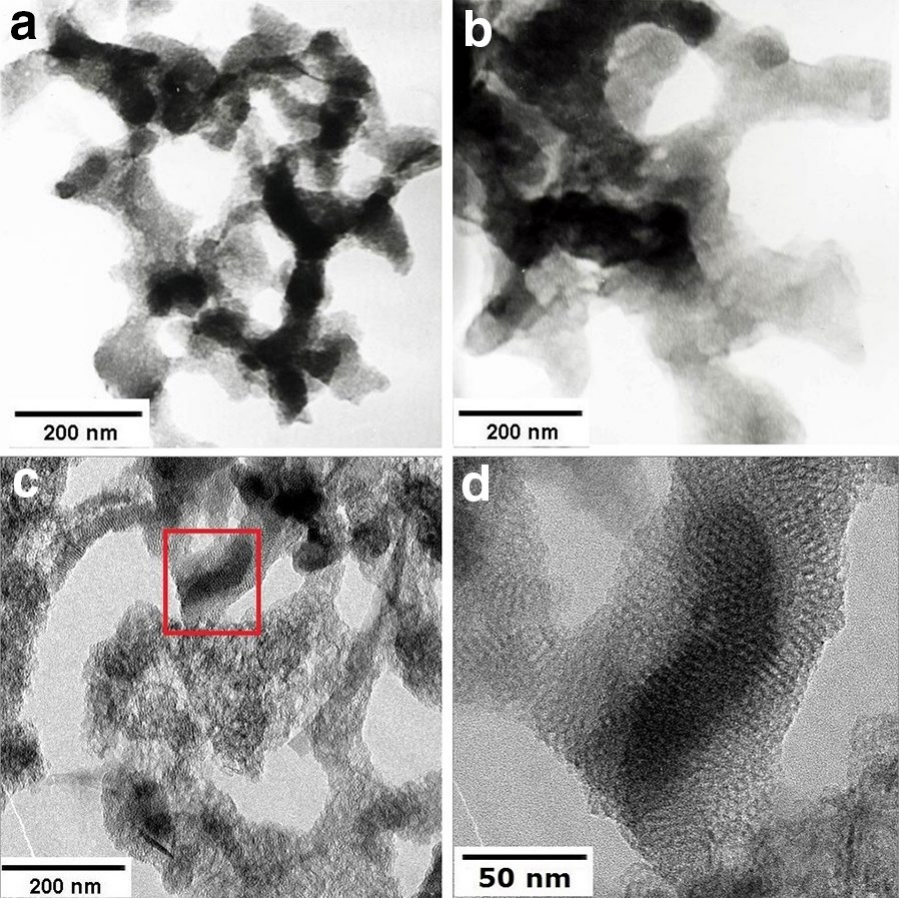
TEM images of POPA-5 (**a**), POPA-6(**b**), POPA-7 (**c**) and TEM images of local area in (**d**) (*red box area*) (**c**)

**Fig. 4:**
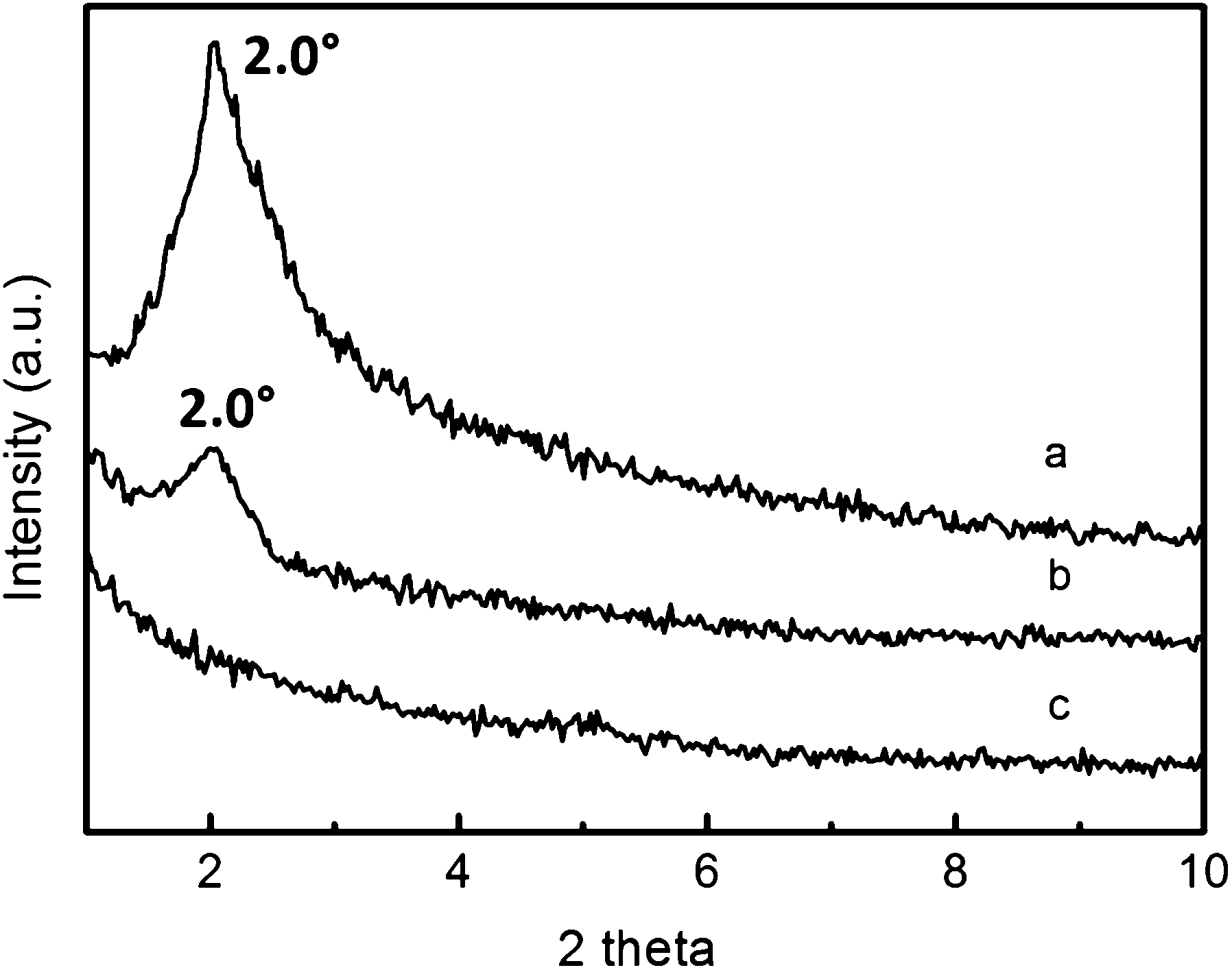
XRD patterns at small angles for POPA-7 (**a**), POPA-6 (**b**), POPA-5 (**c**)

Figure 3a and b are TEM images of the samples with 24-h hydrothermal treatment (POPA-5) and 48-h hydrothermal treatment (POPA-6) respectively. Comparing to the sample without hydrothermal treatment, indistinct mesoporous structure can be found; while rather clearer mesoporous structure (Fig. 3c and d) can be observed in the sample with 96-h hydrothermal treatment (POPA-7). It is obvious that longer time of hydrothermal treatment is advantageous to the formation of uniform ordered mesoporous structure. In addition, XRD patterns showed in Fig. 4 can give detailed repeated structural information of these samples. No obvious peak can be found in the diffractive curve for POPA-5 (Fig. 4c), while a small peak (2θ = 2.0°) which can be attributed to a hexagonal lattice structure appears in the diffractive curve for POPA-6 (Fig. 4b). Furthermore, the intensity of the peak at the same position (2θ = 2.0°) can be increased if the sample is treated for longer time in the sealed hydrothermal reactor, as showed in the diffractive curve for POPA-7 (Fig. 4a). These results can provide mutual confirmation with TEM images above. For POPA-7, the cell size calculated from XRD (Fig. 4a) (hexagonal parameter $a_{0} = 2d_{100} /\sqrt 3$) is about 4.8 nm. The estimated the cell size obtained directly from TEM image (Fig. 3d) is near to 5.0 nm. These results match each other and can give the basic parameter of the obtained mesoporous materials.

Due to the organic–inorganic hybrid structure of POSS-PANI, the dense packing structures of rigid PANI arms are prohibited, instead of loosely packing structures. These structures can convert into stable mesoporous structures by hydrothermal treatments. As we know, the size of octafunctional POSS cage in this experiment is about 1.2 ~ 1.4 nm, which have functional groups in each octant in Cartesian space, either opposite or completely orthogonal to each other. PANI chains that linked in the corner of POSS cages can be regarded as flexible connections among POSS cages. The final morphologies of the POSS-PANI mesostructure after hydrothermal treatments will be affected by many factors, such as conformational energy of flexible PANI chains, van der Waals forces and the interactions between POSS cages, and electrostatic interaction. In POSS-PANI composites, POSS can be regarded as a head-group area, which plays a governing role in works as in building the mesoporous framework and selecting the mesophases. The favored mesoporous structures can permit the area of POSS cage to be closest to the most optimal value, while maintaining favorable packing mode of the hydrophobic PANI chains. In this case, the total energy of the composite tends to minima and the system is most stable.

**Fig. 5:**
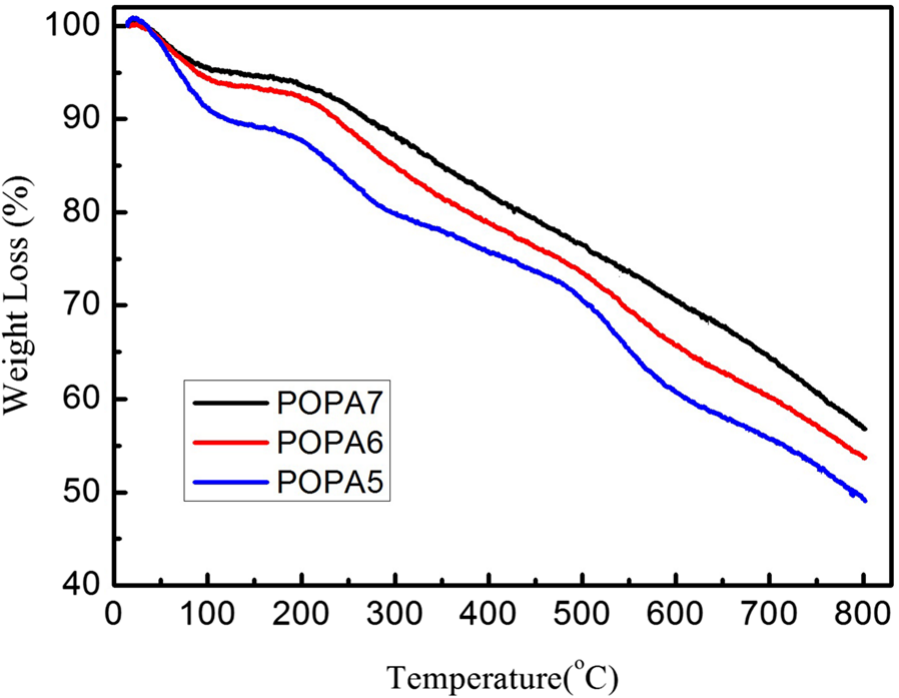
TGA curves of POPA-5 (*blue*), POPA-6(*red*) and POPA-7 (*black*)

TGA curves obtained in atmosphere (Fig. 5) show that the 5 % weight-loss temperatures of POPA-5, POPA-6 and POPA-7 are 65.1, 88.2, 130.1 °C respectively, while the 50 % weight-loss temperatures of the three samples are 508.1, 544.4, 609.4 °C in order. Obviously, longer time of hydrothermal treatment corresponds more regular mesoporous structure, which results in better thermal stability. Porous framework can give many inorganic “rooms” in the composites, which can prevent parts of organic component from degradation into organic volatiles in TGA test. In our experiment, longer time of hydrothermal treatment helps to obtain more regular mesoporous structure, which is advantageous to the improvements in thermal stability.

## Conclusions

In summary, nanostructured POSS-PANI composite fibres have been successfully prepared by using aniline and octaaminophenyl POSS through copolymerization and hydrothermal approaches. The morphology of fibrous POSS-PANI composites can be adjusted by changing POSS concentrations. Further hydrothermal treatment can result in mesoporous structure, which helps to improve its thermal stability. Detailed studies on other properties of nanostructured POSS-PANI composites are now in progress.

## Methods

### Materials and techniques

Aniline, tetramethylammonium hydroxide (Me_4_NOH), phenyltrichlorosilane, triethylamine, ethylacetate, toluene, ammonium peroxydisulfate and fuming nitric acid were bought from Shanghai Experiment Reagent Co., Ltd. and they were of analytical grade.

### Techniques

XRD patterns were obtained by using a Rigaku K/max-γA X-ray diffractometer with a Cu Ka (λ = 1.5415 Å) at the scanning rate of 0.02°/s; Infrared spectra were obtained by using a MAGNA-IR 750 spectrometer. Thermal gravimetric analysis (TGA) was performed on a Netzsch STA-409c Thermal Analyzer under a 50 × 10^3^ mm^3^/min nitrogen or air flow with the heating rate of 10 °C/min; TEM images were obtained from JEOL2010 Transmission Electronic Microscope system. MALDI-TOF data was acquired on a GCT gas chromatography time-of-flight mass spectrometer at the pressure of 0.280 Pa under a certain heating program. NMR spectra were acquired using a Bruker AVANCE 400 spectrometer. The hydrothermal synthesis reactor used in our experiments is made up of two parts: the inner part is a pot with a lid made of polytetrafluoroethylene; the outer part is a stainless-steel kettle, which can play a sealing role by tightening its lid. Hydrothermal synthesis reactor can provide a sealing reaction environment under high temperature and high pressure.

### Synthesis and preparations

**Fig. 6:**
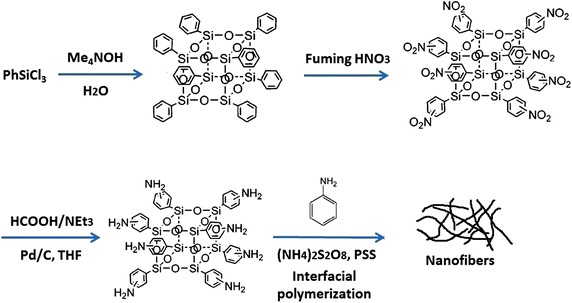
The scheme of experimental approaches to form nanostructured POSS-PANI composites

The synthesis and fabrication strategies of POSS-PANI nanofibre are illustrated in Fig. 6. The synthesis of and octahenyl silsesquioxane (**OPS**) and octa(aminophenyl)silsesquioxane (OAPS) were started from phenyltrichlorosilane (PhSiCl_3_) according to the published ways (Tamaki et al. 2003). Brief description for preparations was as followings: Octaphenyl POSS (OPS) was synthesized via the hydrolysis and condensation of phenyltrichlorosilane and the subsequent rearrangement reaction catalyzed by tetramethylammonium hydroxide, and the obtained product was extracted and dried in vacuum. The nitration of OPS was the carried out in fuming nitric acid to form octa(nitrophenyl) silsesquioxane (ONPS): 50 g OPS was added little by little to 300 mL of fuming nitric acid with stirring in ice water bath. After that, the solution was stirred for 60 min in ice water bath and then at room temperature for a day. After filtration, the solution was poured onto 250 g of ice. Then 10.0 g ONPS, 1.12 g Pd/C, 100 mL THF and 100 mL triethylamine were added into a flask under N_2_ protection. The mixture was heated to 60 °C, and 10.0 mL of 85 % formic acid was added slowly. After 5 h, the THF layer was separated and filtered. The filtrates were combined with 50 mL of ethylacetate and washed by H_2_O. The organic layer was dried and precipitated by addition to hexane. Precipitates (OAPS) were collected by filtration and dried under vacuum for the next preparation. The final yields of OAPS were about 35 %.

Interfacial polymerization was performed in an aqueous/organic biphasic system containing aniline and OAPS mixture dissolved in toluene together with ammonium peroxydisulfate dissolved in an aqueous acid solution. The experiments for interfacial polymerization were carried out in a room with constant temperature and humidity, where the temperature was controlled at (25 ± 1 °C). For sample **POPA-4** with higher POSS concentration, further hydrothermal treatments (PH = 8~9, 120 °C) were carried out after 20-min ultrasonic dispersion. Detailed composite samples are listed in Table 1.
